# Effect of hot water maceration, rehydration, and soft tissue presence on 3D geometry of bone

**DOI:** 10.1007/s12024-024-00845-0

**Published:** 2024-06-15

**Authors:** Martin Bittner-Frank, Andreas Strassl, Ewald Unger, Lena Hirtler, Franz Kainberger, Reinhard Windhager, Francesco Moscato, Emir Benca

**Affiliations:** 1 Department of Orthopedics and Trauma Surgery, Medical University of Vienna, Vienna, Austria; 2 Center for Medical Physics and Biomedical Engineering, Medical University of Vienna, Vienna, Austria; 3 Department of Biomedical Imaging and Image-guided Therapy, Medical University of Vienna, Vienna, Austria; 4 Center for Anatomy and Cell Biology, Medical University of Vienna, Vienna, Austria; 5 Ludwig Boltzmann Institute for Cardiovascular Research, Vienna, Austria; 6 Austrian Cluster for Tissue Regeneration, Vienna, Austria

**Keywords:** Maceration, Rehydration, Bone, Forensic anthropology, Computed tomography, 3D model, Dimensional accuracy

## Abstract

**Purpose:**

In forensic medicine, maceration is often essential for examining bone surfaces, serving purposes such as identifying cut marks, making geometric measurements, and determining the victim’s age. While hot water maceration removes soft tissue effectively, it is known to cause bone surface shrinkage. This raises the question of whether this effect is permanent or if it can be partially reversed through rehydration, considering the presence of soft tissue.

**Methods:**

Computed tomography (CT) scans were conducted on the radii of 20 paired human anatomic forearm specimens. Subsequently, the radii were extracted, macerated in 60 °C water, CT-scanned in an air environment, rehydrated, re-implanted into the forearms, and CT-scanned again.

**Results:**

Maceration resulted in a mean shrinkage of 0.12 mm on the outer bone surface. This shrinkage was nearly fully recoverable for the diaphysis after rehydration and accounting for soft tissue surrounding the bone. In contrast, the epiphysis showed permanent shrinkage, likely due to the loss of small bone fragments. Analysis of the inner bone surface indicated a smaller effect, but with significant standard deviations, especially for the epiphysis, possibly related to the less well-defined nature of the inner bone surface.

**Conclusion:**

The epiphyseal surface of hot water-macerated bone will, on average, be approximately 0.15 mm deflated and cannot retain the original surface. On the other hand, the diaphyseal surface is less affected and can be nearly completely restored after rehydration and accounting for soft tissue surrounding the bone.

**Supplementary Information:**

The online version contains supplementary material available at 10.1007/s12024-024-00845-0.

## Introduction

Over the past three decades, imaging-based forensic anthropology or “virtual anthropology” has garnered significant attention, particularly in the generation of three-dimensional (3D) digital models of human body parts [1–4]. This approach offers several clear advantages, including permanent data storage, data sharing, investigating causes of death when autopsy consent is denied (e.g., in children, depending on the legal system) [5], and improved identification of bone related issues such as fractures [5, 6]. As a result, a variety of imaging tools have been employed sequentially, such as computed tomography (CT) [2, 5–8], magnetic resonance imaging (MRI) [7–9] and optical surface scanning [8, 10].

While CT and MRI offer the advantage of scanning the interior of the human body, these techniques require specially trained technicians, expensive machines primarily located in hospitals, and are not readily available for forensic science [4]. In contrast, optical surface scanners are already in use in traffic injury assessments directly at the accident scene [10]. However, they can only digitize the outer surface, such as the skin and penetrating objects. To investigate the bone itself, soft tissue removal is necessary, e.g., to better reveal healed or recent bone trauma [11]. Additionally, the determination of bone age is also often performed based on geometric measurements of 3D bone models [12, 13], which may differ from the original native bone. Maceration of bone further allows for specimen storage at room temperature, minimizes biological degradation and reduces the risks related to potential pathogenic material.

The most common maceration techniques include mechanical, biological, chemical, heat or hot water methods [14–16]. One significant drawback of hot water maceration, which has been recommended method for investigation of infant, flashed and/or burned remains [17–19], is the partial destruction of DNA, especially when washing detergents are employed [14, 20]. Moreover, hot water maceration has been reported to induce bone shrinkage [21, 22]. While this effect might be negligible in certain cases, such as in determining a fracture location, it could be relevant when identifying cut marks with high precision.

In related fields of anthropology, trauma and orthopedic surgery, virtual 3D bone models are frequently utilized for pre-operative planning. The validation of these models is predominantly carried out using 3D optical surface scans [21, 23–25], due to their high spatial resolution and reproducibility [26]. Notably, it has been observed that 3D models derived from CT images tend to be inflated, whereas those based on MRI are deflated [22]. One potential reason is the partial volume effect (PVE) at the bone-soft tissue interface [27]. However, the critical question arises regarding whether these 3D surface scan models accurately represent the true dimensions. It is conceivable that soft tissue removal through hot water maceration may have caused shrinkage, as has been suggested [21, 22, 28]. This implies that these models might be smaller than the actual ground truth. It is possible that rehydration and accounting for soft tissue presence could, to some extent, restore the original 3D geometry. Nonetheless, to the best of the authors’ knowledge, this combined effect has not yet been investigated.

The objective of the present study was to quantify the effect of hot water maceration on bone dimensions for the distal human radius. The hypothesis was that it would result in a permanent shrinkage of the bone surface. Additionally, it was suspected that rehydration and considering the presence of soft tissue, to some extent, restore the original 3D bone geometry.

## Materials and methods

### Specimens

Fresh-frozen paired anatomic forearm specimens were obtained from 10 body donors (5 male and 5 female, with a mean age of 78 ± 8 years), provided by the Center for Anatomy and Cell Biology, Medical University of Vienna (approved by the Ethics Committee of the Medical University of Vienna). The forearms were amputated at the midsection of the radius and ulna, securely fastened with two laces onto a plastic grid, and then stored in airtight plastic freezing containers at − 20 °C until further use. Hereby, freezing has been shown to cause qualitative changes in cortical bone, e.g., the formation of microcracks [29], whereas no dimensional effect was observed [30], meaning that the overall geometry, the focus of the present study, is likely not significantly affected.

### Bone extraction, maceration and re-implantation

**Fig. 1:**
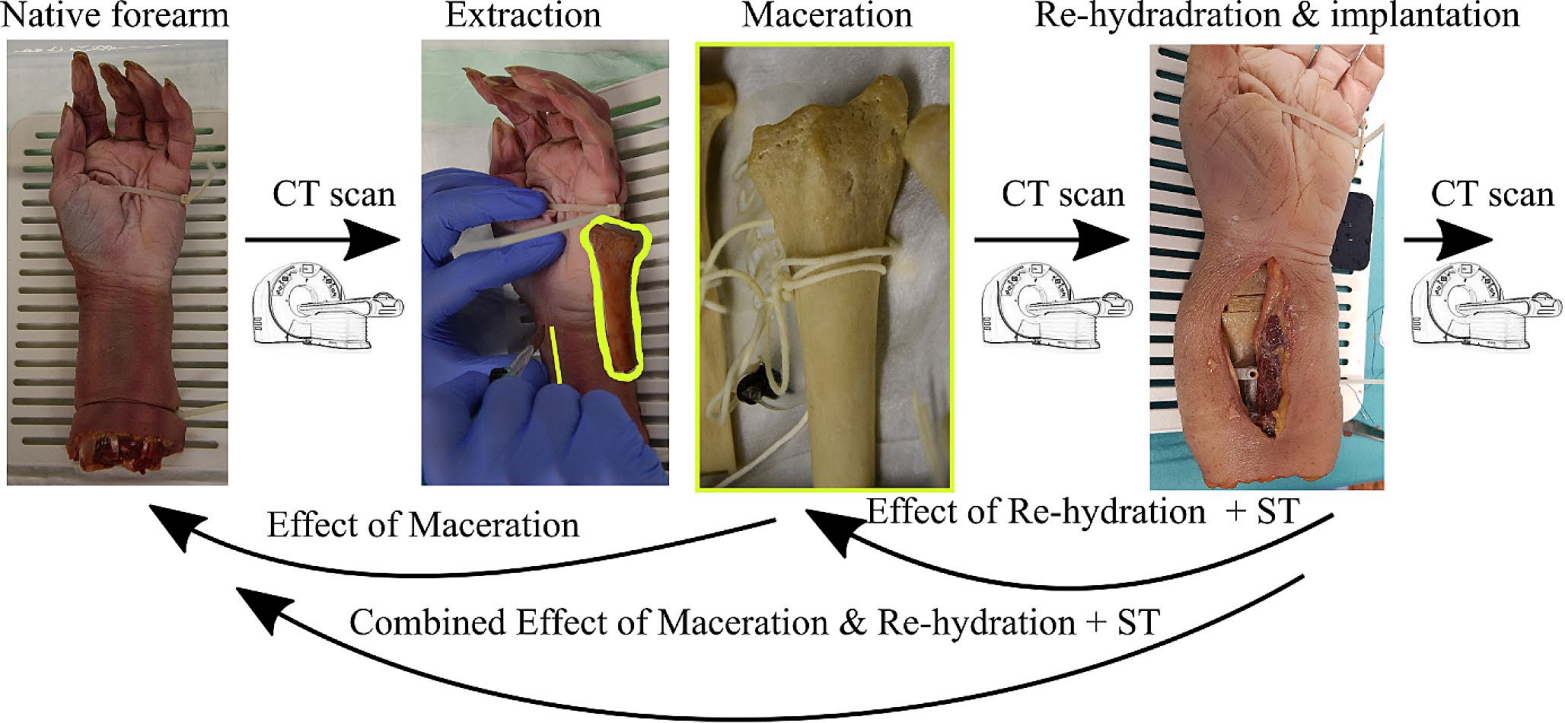
Study overview: Native forearms were initially scanned with CT, the distal radius was extracted with a modified Henry approach, macerated at 60 °C in water for two weeks, scanned with CT in air environment, re-hydrated and implanted, and another CT scan was performed. Determination of the effects of maceration, re-hydration + soft tissue (ST) presence, and the combined effects was performed as indicated with arrows

Anatomic forearm specimens were thawed at 4 °C for 24 h before the initial CT scans were performed (see section CT scanning). Subsequently, the distal radii were extracted using a modified Henry approach (as depicted in Fig. 1). In brief, skin was incised above the M. flexor carpi radialis tendon, and the muscle bellies were mobilized radially. Then, the M. pronator quadratus was incised and all ligaments and tendons attaching to the radius were removed. This allowed the entire distal radius to be mobilized with minimal surgical damage to the surrounding soft tissue. The extracted radii were macerated in water for two weeks at 60 °C to completely remove soft tissue (as shown Fig. 1). Then, the dried radii were subsequently scanned again with CT in air environment. As these radii were used in an additional study related to the 3D model accuracy of simulated bone fractures [31], they were cut using 3D printed osteotomy guides to simulate a dorsally inclined Colles’ fracture. The location of this cut line was determined based on previous work by Baumbach et al. [32], situated at the border between the epiphysis and metaphysis, allowing for individual analysis of the epiphysis. The simulated fractures were then stabilized with epoxy-glass wedges (as seen in Fig. 1 – right) and immersed in a 0.9% NaCl solution for 24 h at room temperature to facilitate rehydration (since it is one of the most widely used rehydration media and has been demonstrated to have no effect onto the bone’s elastic mechanical properties [33]). The goal of the re-hydration was to evaluate the effect of potential tissue swelling. Subsequently, the bone specimens were re-implanted into the forearms at their original anatomical locations. Subcutaneous tissue and skin were closed using suturing materials (V13H, Vicryl, 3 − 0, and EthilonII, polyamide blue monofil 4 − 0, both from Ethicon, Raritan, NJ, USA). Finally, the forearms were once again scanned with CT to assess the combined effect of maceration, rehydration, and the soft tissue presence.

### CT scanning

CT scanning was performed with forearms aligned longitudinally with the SOMATOM Force (Siemens Healthineers AG, Erlangen, Germany), following a standard clinical protocol for the wrist (see Table 1). For the scans of the macerated radii in an air environment, a slightly modified protocol was applied to accommodate differences in the interfaces between bone/soft tissue and bone/air. Hence, scanning at 140 kVp reduced beam hardening, caused by the interface bone/air.

**Table 1:** CT scan protocols and reconstruction settings

Scanned region	Forearm		Bone only
**Scan settings**			
Collimation in mm	64 × 0.6		64 × 0.6
Voltage in kVp	120		140
TCTP^*^ in mAs	300		157
Rotation time in s	1		1
Pitch factor	0.35		0.35
Mean CTDI in mGy	25.4		38.3
**Reconstruction settings**			
Slice thickness in mm	0.40		0.40
Increment in mm	0.40		0.40

* Tube current time product

### Image processing

**Fig. 2:**
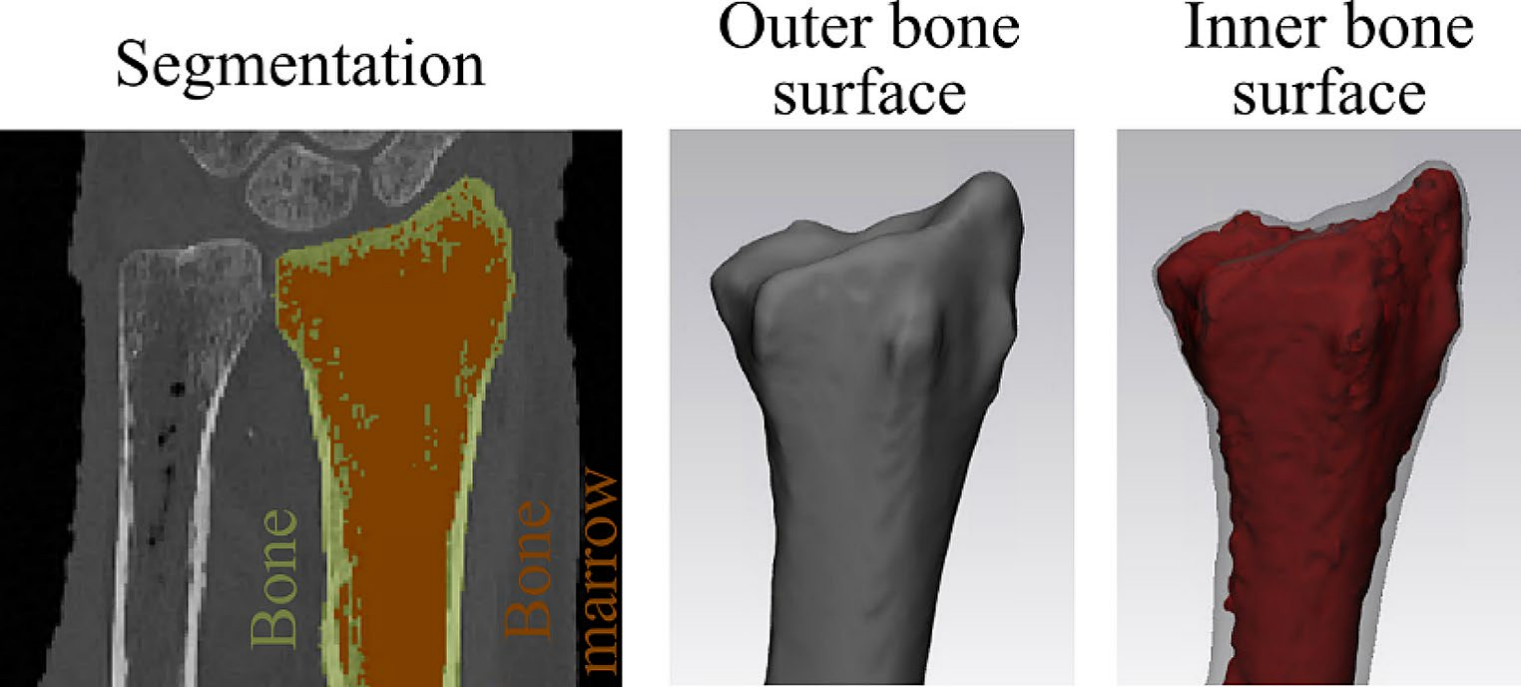
Image processing: CT image series were segmented with a manually determined threshold for the epiphysis and diaphysis and merged into a single bone mask. This mask was filled to obtain the outer bone surface. The inner bone surface was obtained as the subtraction of the filled and segmented bone mask

Image processing was conducted using Mimics Research (V21.0 Materialise NV, Leuven, Belgium). The segmentation of bone involved the application of a single-level threshold for bone, which was manually determined for each, the epiphysis and diaphysis. In brief, a line intensity profile was established on a representative cross-section of the epiphysis and diaphysis, separately (as depicted in Fig. S1). Following the methodology suggested by Hangartner [27], the segmentation threshold was set at the 50% value of the difference between bone and the surrounding soft tissue. This approach allowed for the assessment of the distal border of the diaphysis using the segmentation mask of the diaphyseal bone (as shown in Fig. S1). Next, the masks of the epiphysis and diaphysis were merged into a unified bone mask. The resulting models were filled using the “smart fill” tool, to capture only the outer bone surface (as illustrated in Fig. 2). Additionally, the “bone model” was subtracted from the “filled model” to generate the inner bone surface model. For both the inner and outer bone surface models, 3D parts were generated (setting: “optimal”), following the method proposed by Gelaude et al. [21]. Post-processing of the 3D models involved using the “wrap” function (with one pixel as the “smallest detail” and a half pixel as the “gap closing distance”) and the “smooth” tool (with two iterations and a smooth factor of 0.3, in accordance with [34]). These models were generated for each CT scan, including the initial forearms, the macerated radii in air environment, and the re-implanted forearms. The final models were then exported as Standard Triangle Language (STL) format files.

### Image registration

STL files based on all three different CT scans were imported into 3-matic Research (V 13.0, Materialise NV, Leuven, Belgium) for each specimen individually. Image registration of those models was performed as follows, to determine the effect of maceration, rehydration and the presence of soft tissue, and lastly, the combined effects (see Fig. 1):

- Maceration: Registration of 3D models of macerated radii onto the initial forearm models.
- Re-hydration & soft tissue (ST) presence: Registration of 3D models of the re-implanted radii onto the models of macerated radii scanned in an air environment.
- Combined effects: Registration of 3D models of the re-implanted radii onto those of the initial forearm models.

**Fig. 3:**
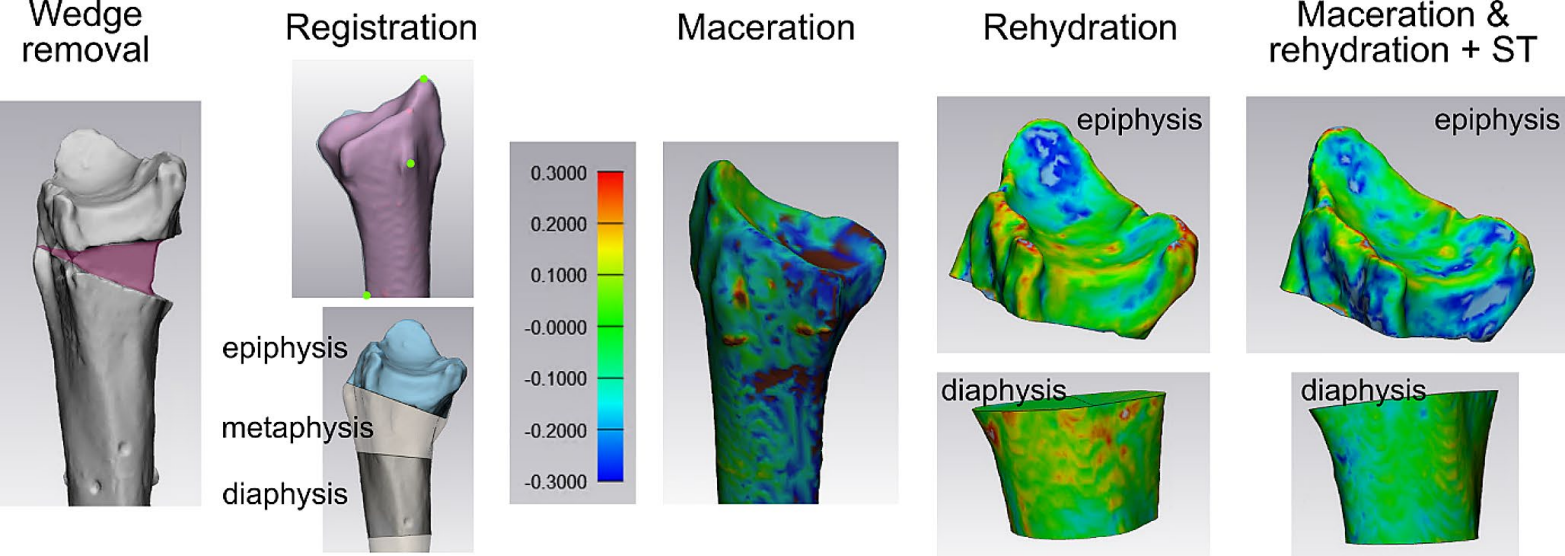
Image registration: The wedge inside the radius which simulates a dorsally inclined Colles’ fracture, was removed. A three-point registration was carried out using the Lister tubercle, the peak of the styloid processus and the most proximal part of the margo interosseus (indicated by the green dots). Subsequently, a global registration was performed to ensure perfect alignment of the epiphysis and diaphysis. Exemplary plots of 3D dimensional deviation are shown for the effect of maceration, rehydration + soft tissue (ST) presence, and the combined effect, for the epiphysis and diaphysis separately. Only for maceration, also the whole distal radius was analyzed. The displayed legend is valid for all plots and given in mm

For these registrations, the “3-point registration” was utilized, involving the selection of the Lister tubercle, the styloid process, and the most proximal point of the interosseous margin (as depicted in Fig. 3). To ensure the convergence of the registration, a “global registration” algorithm was executed with specific parameters (distance threshold: 1.00, 20 iterations, sample percentage 100). Since the final CT scans of the 3D bone fracture models featured a dorsal inclination of the epiphysis, which was stabilized with a wedge, these models required manual processing. In short, the wedge could be easily removed using the trim tool in a perfect lateral view (as shown in Fig. 3). Subsequently applying the “global registration” algorithm was sufficient to achieve a precise alignment of the epiphysis and diaphysis separately (see Fig. 3). The metaphysis was not subjected to further analysis. In a preliminary study involving five models, it was verified that repeated application of the “global registration” resulted in only a minimal decrease in the mean average deviation of less than 0.001 mm, indicating that a single run was sufficient.

### Statistical analysis

Histograms were generated for each specimen and registration in 3-matic Research and exported as text files. A custom-made python script was employed to import these histograms and to compute the mean, standard deviation (std), root mean square error (RMSE), minimum (min), maximum (max), median, and inter-quartile range (IQR) of 3D model dimensional deviation. For enhanced visualization, data was transformed into Probability Density Functions (PDF) and presented as boxplots. Subsequent statistical analysis was conducted using Scipy [35], whereby data from all 20 specimens were pooled and evaluated for each investigated attribute, e.g., maceration, producing ~ 100,000 data points for the dimensional deviations of the 3D surface nets). The shape of the distributions was visualized with histograms and quantified with the Kolmogorov-Smirnov test, demonstrating a non-normal distribution for the majority of data sets. Thus, the Mann–Whitney-U test (for two groups) and a Kruskal-Wallis test (for more than two groups) were utilized with a significance level of α = 0.05 to determine statistically significant differences in dimensions between the models. The p-values were adjusted with the Bonferroni correction for multiple testing.

## Results

**Fig. 4:**
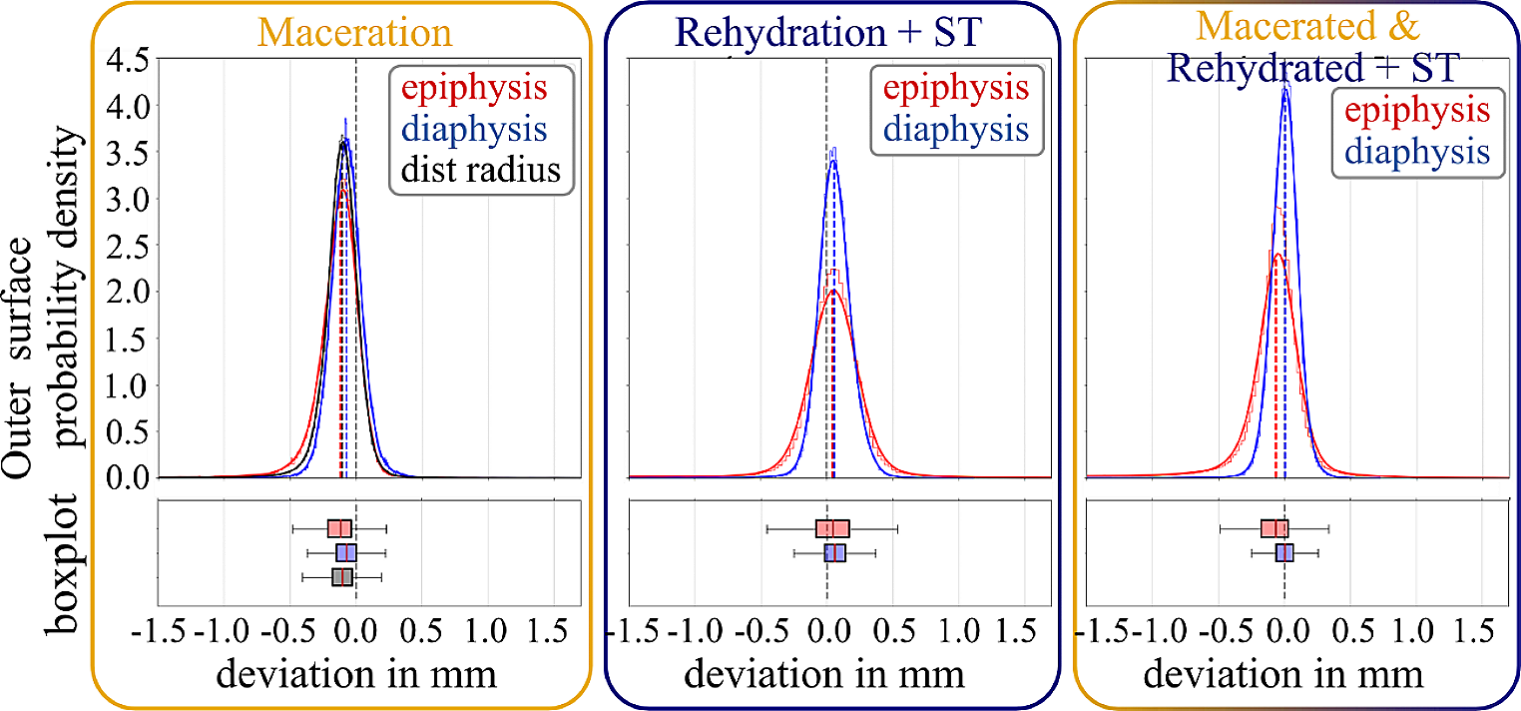
Dimensional deviation of 3D bone models for the outer bone surface following maceration (left), rehydration with re-implantation + soft tissue (ST) presence (middle), and combined effects (right). Probability density functions are shown with histograms and corresponding boxplots (outliers are not plotted, for better visibility due to a large number of data points (∼100,000))

**Fig. 5:**
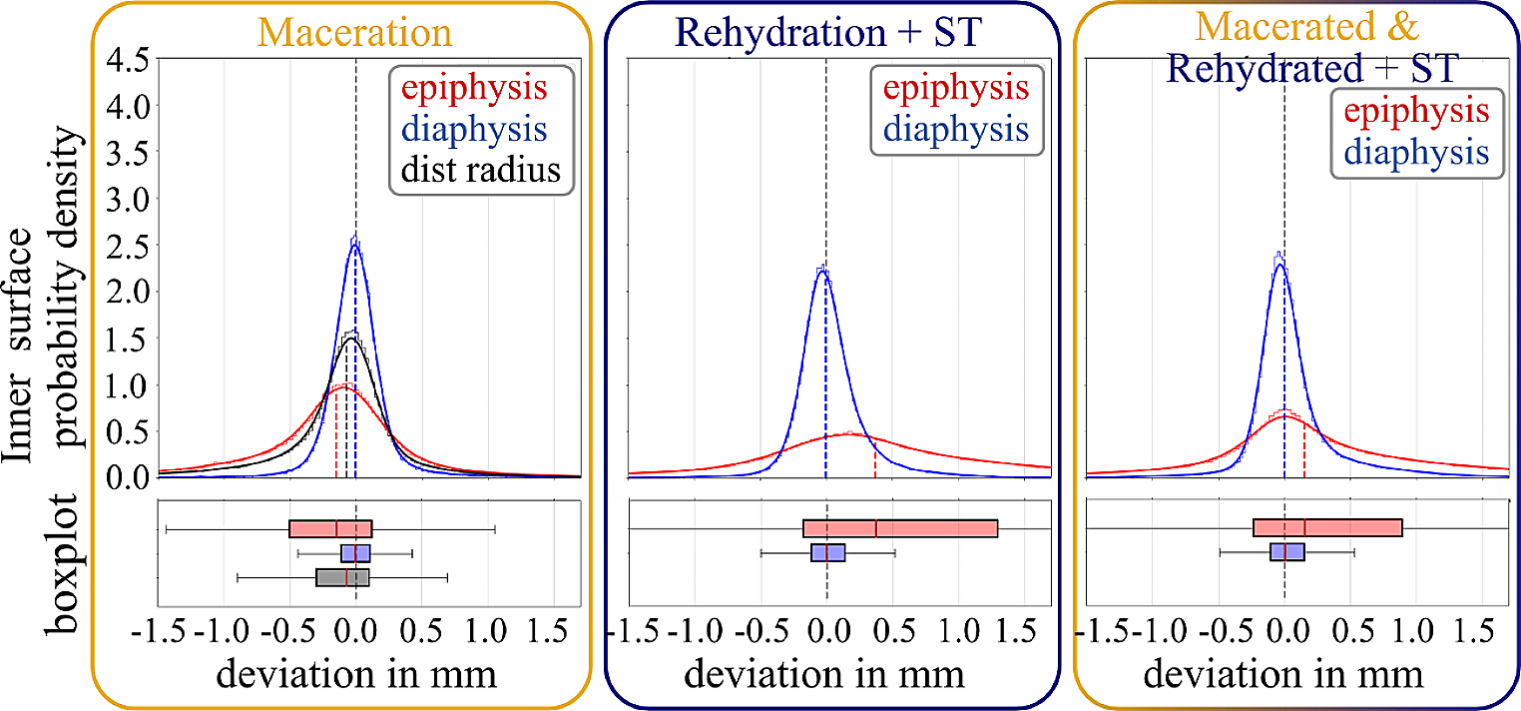
Dimensional deviation of 3D bone models for the inner bone surface following maceration (left), rehydration with re-implantation + soft tissue (ST) presence (middle), and combined effects (right). Probability density functions are shown with histograms and corresponding boxplots (outliers are not plotted, for better visibility due to a large number of data points (∼100.000))

The determination of dimensional deviations of 3D models was assessed in relation to the effects of maceration, rehydration and soft tissue presence, and their combined effect. Separate evaluation was performed for the outer (see Table 2; Fig. 4) and inner bone surface (see Table 3; Fig. 5). The analysis of the outer bone surface revealed a shrinkage of the entire distal radius, with the epiphysis being notably more affected than the diaphysis. Rehydration and reimplantation caused an increase of the median outer bone surface, both of the epiphysis and the diaphysis. However, the standard deviation of the increased 3D geometry after rehydration was larger in the epiphysis than in the diaphysis. The combined effect of maceration, rehydration, and reimplantation showed a permanent shrinkage of the epiphysis, without a significant effect on the diaphysis.

**Table 2:** Dimensional deviation of 3D bone models for the outer bone surface after maceration (top), rehydration with re-implantation + soft tissue (ST) presence (middle), and combined effects (bottom), all units in mm

	mean	std	RMSE	min	max	median	IQR
**Maceration**							
epiphysis	-0.15	0.26	0.30	-4.48	1.53	-0.12	0.18
diaphysis	-0.07	0.13	0.15	-1.18	1.61	-0.07	0.15
distal radius	-0.12	0.21	0.25	-4.59	3.06	-0.11	0.15
**Rehydration + ST**							
epiphysis	-0.08	0.84	0.85	-7.99	6.36	0.05	0.25
diaphysis	0.05	0.25	0.26	-5.66	1.11	0.06	0.16
**Maceration &** **Rehydration + ST**							
epiphysis	-0.24	0.80	0.84	-8.05	2.42	-0.07	0.21
diaphysis	-0.01	0.24	0.24	-5.77	0.72	0.00	0.13

Std: standard deviation, RMSE: root mean squared error, min: minimum, max: maximum, IQR: inter-quartile-range

**Table 3:** Dimensional deviation of 3D bone models for the inner bone surface after maceration (top), rehydration with re-implantation + soft tissue (ST) presence (middle), and combined effects (bottom), all units in mm

	mean	std	RMSE	min	max	median	IQR
**Maceration**							
epiphysis	-0.26	0.87	0.90	-5.96	6.47	-0.15	0.62
diaphysis	0.00	0.29	0.29	-2.66	2.77	0.00	0.22
distal radius	-0.17	0.71	0.73	-5.93	6.48	-0.07	0.40
**Rehydration + ST**							
epiphysis	0.72	1.83	1.97	-6.32	11.44	0.37	1.47
diaphysis	0.03	0.37	0.38	-4.98	3.96	0.00	0.25
**Maceration &** **Rehydration + ST**							
epiphysis	0.40	1.60	1.65	-6.76	9.74	0.15	1.13
diaphysis	0.08	0.47	0.48	-4.52	5.55	0.00	0.26

Std: standard deviation, RMSE: root mean squared error, min: minimum, max: maximum, IQR: inter-quartile-range

The inner epiphyseal bone surface showed a shrinkage following maceration, again, without significant effect on the diaphysis (see Table 3; Fig. 5). Similarly, there was only a minor increase in the inner diaphyseal bone surface following rehydration and reimplantation. In contrast, there was a significantly more substantial increase of the inner epiphyseal bone surface following rehydration, although the standard deviation of 3D geometry deviation was notably large. Surprisingly, there was even a permanent increase in the inner bone surface of the epiphysis for all combined effects, while the diaphysis was nearly unaffected.

## Discussion

While medical imaging technologies have significantly changed the field of forensic pathology, bone maceration remains an established method in this field.

Maceration of distal radii in hot water (at 60 °C) caused an average shrinkage of (− 0.12 ± 0.21) mm of the outer bone surface (see Table 2; Fig. 4). Hereby, the epiphysis was significantly more affected than the diaphysis (− 0.15 mm vs. −0.07 mm, on average). One possible explanation could be the significantly thinner cortex of the epiphysis. Consequently, maceration might have led to a more pronounced increase in porosity and, potentially, loss of small bony fragments. As a result, the standard deviation of the dimensional deviation in the epiphysis was higher than in the diaphysis (± 0.26 mm and ± 0.13 mm, respectively). Surprisingly, there was no relevant effect of maceration on the inner bone surface of the diaphysis (see Table 3; Fig. 5), whereas there was an average shrinkage of (− 0.26 ± 0.87) mm of the inner surface of the epiphysis. It’s important to note that determining the inner surface of the epiphysis is considerably more susceptible to errors due to the low contrast in Hounsfield unit (HU) values between trabecular bone and bone marrow. In contrast, the inner structure of the diaphysis is primarily composed of cortical bone (with some trabecular remnants), providing a much clearer contrast in relation to bone marrow. Nevertheless, the endocortical region is still more porous and known to present challenges in image segmentation [36]. In accordance, standard deviation of the dimensional deviation of the inner diaphyseal surface was much higher (± 0.29 mm) than the outer surface (± 0.13 mm). The inner epiphyseal surface indicated an even more prominent standard deviation of ± 0.87 mm, which might be attributed to loss of loosely connected trabecular bone, as reported previously [21].

The findings presented here align with previous observations of bone shrinkage after boiling. For example, a previous study reported a shrinkage of (-0.16 ± 0.18) mm in human tibiae following boiling [22]. Similarly, Gelaude et al. [21] found an average shrinkage of − 0.49 mm after boiling human femora. It’s worth noting that these variations may be attributed to differences in CT image acquisition, with the voxel size being 1 mm × 1 mm × 1 mm in [21], compared to 0.39 mm × 0.39 mm × 0.63 mm in [22] and 0.39 mm × 0.39 mm × 0.40 mm in the present study. When considering the observed values relative to voxel size, it becomes apparent that hot water maceration results in a relative shrinkage of approximately 1/3 to 1/2 voxel, as determined by CT.

Previous studies [21, 22, 25] focused only on the effect of soft tissue removal, without accounting for the effect of rehydration and soft tissue presence surrounding bone. In contrast, Oka et al. [37] determined directly the effect when a soft tissue phantom was surrounding the bone and found an average increase of 0.06 mm of outer 3D model surface. In the current study, radii were rehydrated in 0.9% NaCl solution, and re-implanted into the forearm specimens to account for soft tissue presence. Hereby, a median shift of (0.05 ± 0.25) mm was determined in the outer epiphysis (skewed towards negative values, mean: −0.08 mm). Similarly, the outer diaphysis was inflated by (0.06 ± 0.16) mm (and only minorly skewed, mean: 0.05 mm). These findings coincidence closely to the pure effect of soft tissue presence by Oka et al. [37]. Interestingly, Finlay and Hardie [28] showed that rehydration of bovine femora (after dehydration at 40 °C in an oven) increased the bone thickness by ∼3% in the radial direction. Assuming the radii as hollow cylinders with ∼15 mm outer diameter of the diaphysis and an evenly distributed increase of the inner and outer surface should lead to ∼0.11 mm increase of the outer contour. This value is much higher than the observed value in the present study (0.05 mm on average) and might be the consequence of different treatments, namely dehydration at 40 °C in an oven [28], versus maceration in water at 60 °C and (potential) consequent dehydration in air environment.

The combined effect of maceration, rehydration, and accounting for soft tissue presence indicated an almost negligible effect onto the outer diaphysis of (− 0.01 ± 0.24) mm, compared to a permanent decrease of (− 0.24 ± 0.80) mm observed for the epiphysis. As mentioned previously, the surface of the diaphysis is, due to lower porosity and higher proportion of thick dense bone tissue, easier to define than the thin cortical shell of the epiphysis. As a result, it is speculated that the previously observed shrinkage after maceration is primarily attributed to the omission of soft tissue presence rather than solely due to dehydration. While the investigated effect of rehydration has little to no significance in forensic anthropology, it might of interest for other biomedical disciplines, such as biomechanics or additive manufacturing.

In the field of medical pre-operative planning, it is essential to know that 3D models generated from CT scans will be inflated by ∼0.10 mm, compared to the native bone. However, since the required accuracy of these models is 0.50 mm [37], it can be assumed that this effect can be neglected for most trauma and orthopedic surgeries. Potentially, the accuracy of the obtained 3D models might be dependent on the operator, e.g., the level of experience. However, in a recently published paper the inter- and intra-operator variability of image registration was performed on the same distal radius fracture models [31] with different levels of experience of 5 operators, ranging from novice to several years of experience in image processing. Hereby, an excellent inter- and intra-operator reliability for the dimensional deviation in terms of intra-class correlation (ICC) was found with 0.92 and 0.75, respectively. Measurement errors of the 3D geometry of bone models are likely to be negligible, since the image processing protocol was performed with an established software (Mimics and 3-matic (Materialise NV)), using well established protocols from literature for 3D part generation [21] and post-processing of the 3D models [34]. In the current study, the effect of maceration was determined utilizing CT scanning, but not 3D surface scanning, due to technical limitations of scanning wet surfaces. Further, the CT scan protocol was slightly altered between forearms and macerated bones, because of differences in the interface to surrounding tissue/air. This limitation is, however, partly weakened as a parallel work demonstrated only a minor effect of the reconstruction kernel onto detectability of small features [data currently under review]. The osteotomy of the distal radius simulating Colles’ fracture might have contributed to limited trabecular loss, especially in the epiphysis, exacerbating determination of the inner bone surface. Additionally, CT scanning after the re-implantation of bones revealed the presence of some air bubbles inside the epiphysis. These bubbles could potentially lead to errors in the segmentation and the process of generating 3D models, particularly for the inner surface. A further limitation is that the analysis was performed only for a specific bone. Although the results of the diaphyseal radius might be partially transferable to other long bones, the epiphysis of other bones might significantly vary from that of the radius (e.g., the proximal femur) and requires separate analysis for a wider generalization of the presented findings. Maceration was only performed with hot water, while other maceration techniques, such as mechanical, biological, chemical, and thermal were not considered.

## Conclusion

In summary, 3D models obtained from bones after hot water maceration are indeed approximately 0.12 mm smaller than the original bone. Consequently, if dimensional measurements of bone are required at high precision, e.g., evaluation of cut marks in forensic sciences, it might be important to be aware of the aforementioned significant difference. As such, it is essential to account these dimensional deviations in such cases, where high precision geometric measurements are required.

## Key Points

1. Hot water maceration of bone causes shrinkage of 0.12 mm of its surface.
2. Shrinkage is recoverable in the diaphyseal, but permanent in the epiphyseal bone.
3. Soft tissue presence and rehydration are essential for true bone dimensions.

## Electronic supplementary material

Below is the link to the electronic supplementary material.

Supplementary Material 1
