# Supplementary material for: Effect of hot water maceration, rehydration, and soft tissue presence on 3D geometry of bone
Source: Forensic Sci Med Pathol. 2024 Jun 15;21(1):98–106. doi: 10.1007/s12024-024-00845-0 (PMC11953162; doi:10.1007/s12024-024-00845-0)
Supplement: Supplementary file 1 — Supplementary Material 1 [file 12024_2024_845_MOESM1_ESM.docx]

# Supplementary


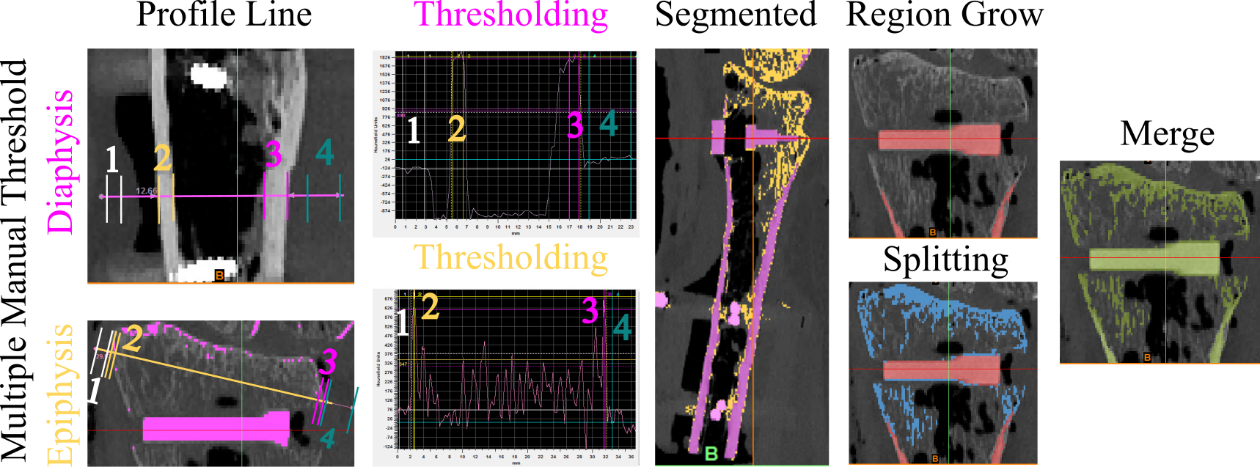


**Fig. S 1**: Image segmentation with a multi threshold for the epiphysis and diaphysis, based on a line intensity profile. As suggested by Hangartner [27], a 50% threshold between bone and soft tissue was selected for segmentation. Hereby, the diaphysis and epiphysis were analyzed separately. Then, overlapping regions were isolated and both regions merged into a single bone mask.
